# Impact of single annual treatment and four-monthly treatment for hookworm and *Ascaris lumbricoides*, and factors associated with residual infection among Kenyan school children

**DOI:** 10.1186/s40249-017-0244-z

**Published:** 2017-02-09

**Authors:** Stella Kepha, Charles S. Mwandawiro, Roy M. Anderson, Rachel L. Pullan, Fred Nuwaha, Jorge Cano, Sammy M. Njenga, Maurice R. Odiere, Elizabeth Allen, Simon J. Brooker, Birgit Nikolay

**Affiliations:** 1 School of Public Health, Makerere University College of Health Sciences, Kampala, Uganda; 2 Eastern and Southern Africa Centre of International Parasite Control, Kenya Medical Research Institute (KEMRI), Nairobi, Kenya; 3 London Centre for Neglected Tropical Disease Research, Department of Infectious Disease Epidemiology, School of Public Health, Imperial College London, London, UK; 4 London School of Hygiene and Tropical Medicine, London, UK; 5 Centre for Global Health Research, KEMRI, Kisumu, Kenya; 6 KEMRI-Wellcome Trust Research Programme, Nairobi, Kenya

**Keywords:** School-based deworming, Soil-transmitted helminths, Albendazole, School children, Kenya

## Abstract

**Background:**

School-based deworming is widely implemented in various countries to reduce the burden of soil-transmitted helminths (STHs), however, the frequency of drug administration varies in different settings. In this study, we compared the impact of a single annual treatment and 4-monthly treatment over a follow-up among Kenyan school children, and investigated the factors associated with residual infection.

**Methods:**

We performed a secondary analysis of data from a randomized trial investigating whether deworming for STHs alters risk of acquiring malaria. Children received either a single treatment or 4-monthly albendazole treatments were followed longitudinally from February 2014 to October 2014. The relative impact of treatment and factors associated with residual infections were investigated using mixed-effects regression models. Predisposition to infection was assessed based on Spearman’s rank and Kendall’s Tau correlation coefficients.

**Results:**

In the 4-monthly treatment group, the proportion of children infected with hookworm decreased from 59.9 to 5.7%, while *Ascaris lumbricoides* infections dropped from 55.7 to 6.2%. In the single treatment group, hookworm infections decreased over the same time period from 58.7 to 18.3% (12.6% absolute difference in reduction, 95% *CI*: 8.9–16.3%), and *A. lumbricoides* from 56.7 to 23.3% (17.1% absolute difference in reduction, 95% *CI*: 13.1–21.1%). There was strong evidence for predisposition to both STH types. Residual hookworm infection among children on 4-monthly treatment were associated with male sex and baseline nutritional status, whereas *A. lumbricoides* infection was associated with individual and school-level infection at baseline, latrine cleanliness at schools.

**Conclusions:**

This study found that 4-monthly treatment w more effective than single annual treatment. Repeated treatments led to dramatic reductions in the intensities of STHs, but did not completely clear infections among school children in Kenya, a presumed reflection of reinfection in a setting where there is ongoing transmission.

**Electronic supplementary material:**

The online version of this article (doi:10.1186/s40249-017-0244-z) contains supplementary material, which is available to authorized users.

## Multilingual abstracts

Please see Additional file 1 for translation of the abstract into the five official working languages of the United Nation.

## Background

Soil-transmitted helminths (STHs: *Ascaris lumbricoides*, *Trichuris trichiura*, and hookworms) are some of the most common parasites that infect humans [1–3]. Fortunately, these parasites can be readily treated using single-dose, safe, and often donated benzimidazole drugs, including albendazole and mebendazole. Children, however, are at a higher risk of becoming reinfected rapidly and previous studies have suggested that children with higher infection intensities at baseline also reacquire worms at higher rates [4–7].

**Fig. 1:**
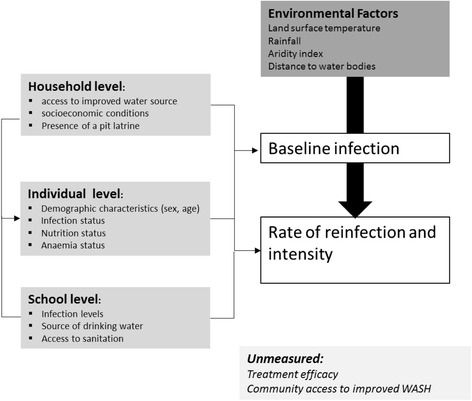
Analytical framework. Overall distribution and occurrence of STHs are influenced by environmental factors that ensure the survival of infectious stages. Factors in the immediate environment where children live (both at the household and school levels) also influence rate of STH infection and reinfection. Intrinsic factors such as nutritional status, age, and sex-related behaviours play a role in infections. Having an initial STH infection predisposes a child to having another infection

The impact of treatment against STHs depends on a variety of factors, including therapeutic efficacy of drugs against individual species [8], the frequency at which treatment is given [9], individual host factors [10–13], and the underlying intensity of parasite transmission [14]. In turn, the intensity of transmission is influenced by factors acting within and outside the host (see Fig. 1), including environmental conditions that influence the survival and development of STH free-living stages [1, 3] and socioeconomic factors, such as access to water and hygiene sanitation (WASH), which influence rates of exposure to infective stages.

In an effort to further understand the factors that determine the effectiveness of anthelmintic treatment, we conducted a secondary analysis of data from an individually randomized trial, which had the primary purpose of investigating whether anthelminthic treatment altered the risk of malaria infection among Kenyan school children [15]. Recruited children were randomized to receive either repeated (every 4 months) treatment or a single annual treatment, and were followed longitudinally for 15 months. At final follow-up, it was found that 6% of children in the 4-monthly treatment group were still infected with either hookworm or *A. lumbricoides* infection despite receiving up to four rounds of albendazole treatment. Motivated by this observation, the aims of the present analysis were to (i) quantify the impact of repeated versus single annual treatment on levels of hookworm and *A. lumbricoides* infection, (ii) investigate any evidence for predisposition to infection among repeatedly treated children, and (iii) identify factors associated with residual infections at the 15-month follow-up point.

## Methods

### Study design and procedures

Full details of the study population, design, and outcomes were previously described Kephaet al. [15]. The trial was conducted in 23 purposively selected schools between January 2013 and October 2014 in Bumula District, Bungoma County, Western Kenya. All children in classes 1–6 (typically aged 5–15 years) with informed consent from a parent or legal guardian were asked to provide a single stool sample, which was examined in duplicate for the presence of hookworm, *A. lumbricoides*, and *T. trichiura* eggs using the Kato-Katz method.

The trial originally recruited 1 505 children with detectable STH infections and 841 uninfected children [15]. Enrolled children were randomly assigned to one of two treatment groups, either (i) a single dose of 400mg albendazole (Zentel®, GlaxoSmithKline South Africa, Cape Town) at baseline and a single 250mg dose of vitamin C (Cosmos Limited, Nairobi) at 4, 8, and 12 months, or (ii) a single dose of 400mg albendazole every 4 months for 12 months. Cross-sectional surveys investigating the participating children’s infection status and intensity (egg counts) were carried out at baseline, and 7, 11, and 15 months.

### Anthropometric and nutritional status data

At baseline, each child’s weight was measured to the nearest 0.1kg using an electronic balance, and height was measured to the nearest 0.1cm using a portable fixed base stadiometer. Hemoglobin concentration was assessed using a hemoglobin photometer (HemoCue® Hb 201^+^ System, Ångelholm, Sweden). Anthropometric indices for nutritional status at baseline included z-scores of height for age (HAZ), weight for age (WAZ), and body mass index for age (BMIZ), and were calculated using the World Health Organization (WHO) AnthroPlus software Stata macro for children aged 5–19 years [16]. Age was self-reported as it was logistically difficult to collect exact birth dates, and because there were doubts over precision a mid-year age was assumed. Children were classified as stunted, underweight, or thin if their HAZ, WAZ, and BMIZ scores were below -2 standard deviations from the reference median. To investigate potential influences of assuming the mid-year age, we conducted a sensitivity analysis using the lowest and highest possible exact ages of children (e.g. 8.0 and 8.9 for the midpoint age of 8.5).

### Household data

At enrolment, a household questionnaire was administered to parents/guardians to collect information on the construction materials of their houses (wall, floor, and roof); sources of fuel; mobile phone ownership; and level of education of the household head. These factors were used to generate a wealth index based on a principal component analysis (PCA) [17], which was then divided into two groups (poor and less poor) based on median (see Additional file 2: Table S1). Household-level access to water and sanitation was assessed by direct observation and included information on source of drinking water and presence of a pit latrine.

### School data

School-level data on WASH were collected by interviewing the head teacher or deputy head teacher, and by visual inspection, using questionnaires and checklists developed for a previous study in Kenya [18]. Conditions of school sanitation facilities were assessed based on observed ‘cleanliness’ of the latrine, presence of visible feces, excessive smell, and excessive flies combined by PCA (Additional file 2: Table S1). The ratio of children per latrine was determined as an indicator for access to sanitation, and was calculated by dividing the number of enrolled children by the number of latrines available in the school. We also asked the head teacher about the source of drinking water, availability of water, and availability of soap and handwashing facilities near latrines.

School locations were mapped using a handheld eTrex 20 global positioning system (Garmin Ltd., Olathe, KS, USA). Estimates of land surface temperature, aridity index, enhanced vegetation index, elevation, and normalized difference vegetation index were determined for each school after averaging the values of covariates within 1-km catchment area. A detailed description of sources and the pre-process of environmental data are provided in Additional file 2, Section 2.

### Statistical analysis

#### Quantification of treatment impact

The impact of repeated (4-monthly) treatment was assessed based on data from 579 children, who were infected at baseline with any STH and presented at the 15-month follow-up point. The proportions of children with hookworm, *A. lumbricoides*, and *T. trichiura* infections together with 95% confidence intervals (*CI*s) were calculated at baseline and at the 15-month follow-up point using binomial regression analysis. Clustering of infection by school was taken into account by estimating clustered robust standard errors as children in the same school may have similar risk of STH infection compared with children from different schools. Intensity of infection was measured as eggs per gram (epg) of feces, and the arithmetic mean epg with 95%*CI*s was estimated using negative binomial regression taking school clustering into account. Reductions in infection levels between baseline and the 15-month follow-up point were investigated using a mixed-effects logistic model (for infection status) and a negative binomial regression model (for intensity of infection), with the individual outcomes at the two time points treated as a repeated measures outcome and a random intercept for schools. Additionally, relative reductions were calculated as the percentage difference between the proportions of children infected, or the mean intensity at baseline and at the 15-month follow-up point.

To assess the efficacy of repeated (4-monthly) treatment in relation to a single annual treatment (as delivered through the Kenyan national deworming program), treatment success was also quantified among the single treatment group (562 children) using the same statistical methods as outlined above. To assess comparability between children in the two study arms, summary statistics were calculated for all individual, household, and school-level characteristics pertaining to the children, by treatment group. To quantify the added benefit of repeated (4-monthly) treatment compared to standard deworming, absolute differences in the proportion of residual infections at 15 months between the treatment groups were estimated together with 95% *CI*s using *prtest* in Stata. (STATA Corp, College Station, Texas, USA) This approach was chosen as, due to trial randomization, any difference in baseline infections between the treatment groups would be due to chance. Therefore, a difference in reduction over 15 months can be directly observed in residual infections.

#### Predisposition to infection

Predisposition to infection was investigated based on data collected from 1,141 children in the 4-monthly treatment group at baseline and at the 7-, 12-, and 15-month follow-up points. Two non-parametric rank correlation tests (Spearman’s rank and Kendall’s Tau) were used for the pairwise comparison of infection intensities in children in all observation rounds from baseline to the 15-month follow-up point for hookworm and *A. lumbricoides*. For both tests, we rejected the null hypothesis (absence of predisposition) if *P*-values were ≤ 0.05.

#### Factors associated with residual infection following repeated treatment

Residual infections assessed 3 months after the delivery of the fourth treatment dose provide an indication of either rapidly occurring reinfections or a lack of parasite clearance after treatment. The analysis was based on 579 children in the 4-monthly treatment group; outcomes were the proportion and intensities of hookworm and *A. lumbricoides* infections at the last follow-up (15 months after the baseline assessment). Risk factors associated with hookworm and *A. lumbricoides* infections at 15 months were investigated using mixed-effects regression models (logistic regression for infection status and negative binomial regression for intensity) with a random school intercept. All statistical models were adjusted for baseline individual infection (infection status for logistic regression and intensity of infection for negative binomial regression). We first investigated associations by including one covariate at a time (unadjusted analysis). Variables with significant associations (*P* ≤ 0.05, based on a likelihood ratio test) were combined into an adjusted regression model, which was then reduced to a final model using a backwards variable selection approach, eliminating one variable at a time based on the highest *P*-value and retaining only variables in which *P* ≤ 0.05 (adjusted analysis).

### Ethical considerations

The study was approved by the Kenya Medical Research Institute Ethics Review Committee (SSC 2242), the London School of Hygiene and Tropical Medicine (LSHTM) Ethics Committee (6210), and the Makerere School of Public Health Institutional Review Board (IRB00005876).

Written informed consent was obtained from a parent or guardian of each child, and assent was sought from children before enrolment into the study. A questionnaire was administered to parents/guardians to collect information on household socioeconomic characteristics.

## Results

### Characteristics of the children under study, and information on their households and schools

Characteristics of 1 141 children who were infected with any STH at baseline and were successfully followed up at 15 months are summarized in Table 1. The majority of infections were due to hookworm or *A. lumbricoides,* and only 1% of children were infected with *T. trichiura*. At baseline, a fifth of the children had a hookworm-*A. lumbricoides* coinfection, while at the 15-month follow-up point, only 4% had this coinfection. All children who were coinfected at 15 months were from the single annual treatment group.

**Table 1:** Characteristics of children who were infected with any STH at recruitment and presented at the 15-month follow-up point (*n* =1 141), by treatment group

	Treatment group	
Characteristics^a^	Annual treatment (*N* = 562)	Four-monthly treatment (*N* = 579)
*Characteristics*		
Sex, male	54.8 (308/562)	53.4 (309/579)
Age, years	10.3 (2.5)	10.4 (2.5)
Thin	12.5 (70/562)	11.1 (64/579)
Stunted	27.2 (153/562)	27.3 (158/579)
Underweight^b^	6.12 (18/294)	6.25 (19/298)
Hemoglobin, g/dL	12.3 (1.3)	12.3 (1.4)
Anemia	36.0 (194/539)	40.7 (227/558)
STH infection		
Hookworm	58.7 (330/562)	59.9 (347/579)
*Ascaris lumbricoides*	55.7 (313/562)	56.7 (328/579)
*Trichuris trichiura*	1.6 (9/562)	1.2 (7/579)
Any STH infection	100 (562/562)	100 (579/579)
STH infection intensity (epg)		
Hookworm	106 (66–185)	180 (98–330)
*A. lumbricoides*	3 234 (2 539–4 119)	2 605 (1 968–3 449)
*Household characteristics*		
Socioeconomic status		
Poor	64.3 (342/532)	64.1 (356/555)
Less poor	35.7 (190/532)	35.9 (199/555)
Water source		
Non-improved drinking water	6.7 (36/538)	3.5 (20/558)
Improved drinking water	93.3 (502/538)	96.4 (538/558)
Floor		
Mud	95.2 (513/539)	94.1 (526/559)
Cemented	4.8 (26/539)	5.9 (33/559)
Pit latrine		
No	11.3 (61/539)	11.6 (65/560)
Yes	88.7 (478/539)	88.4 (495/560)
*School-level characteristics*		
Baseline school prevalence		
Hookworm		
< 33%	31.3 (176/562)	36.8 (213/579)
34–45%	35.6 (200/562)	32.5 (188/579)
> 45%	33.1 (186/562)	30.7 (178/579)
*A. lumbricoides*		
<27%	35.4 (199/562)	38.8 (225/579)
28–30%	26.2 (147/562)	26.9 (156/579)
> 40%	38.4 (216/562)	34.2 (198/579)
School mean infection intensity		
Hookworm		
6–35	31.3 (176/562)	36.8 (213/579)
44–90	35.6 (200/562	32.5 (188/579)
102–453	33.1 (186/562)	30.7 (178/579)
*A. lumbricoides*		
366–913	34.3 (193/562)	41.5 (240/579)
914–2497	29.7 (167/562)	29.0 (168/579)
> 2497	35.9 (202/562)	29.5 (171/579)
Latrine cleanliness		
Clean	47.8 (269/562)	53.4 (309/579)
Dirty	26.3 (148/562)	26.8 (155/579)
Very dirty	25.8 (145/562)	19.9 (115/579)
Children per latrine		
< 50:1	42.9 (241/562)	42.0 (243/579)
> 50:1	57.1 (321/562)	58.0 (336/579)

^a^Data are proportions (N/n), unless otherwise stated

^b^Provided for children aged 5–10

The mean age of enrolled children at baseline was 10.4 years (standard deviation [SD]: 0.05), range 5–15 years. A quarter of the children were classified as stunted and a tenth as thin. In the children’s households, WASH characteristics were generally homogenous: 88% had a pit latrine and 95% derived drinking water from a covered source.

In the children’s schools, WASH conditions were generally poor. All 23 schools had ordinary pit latrines. Most schools (18/23) had functional pit latrines, but in one school all the pit latrines were in a deplorable state. Whilst five schools had handwashing facilities near the latrine, only four facilities contained water. Two schools reported soap availability, but did not have handwashing facilities near the toilet. Boreholes were the main source of drinking water at the schools (21/23); four schools reported not having access to drinking water for 2–3 months a year.

### Reduction of infections after repeated or single treatment

**Fig. 2:**
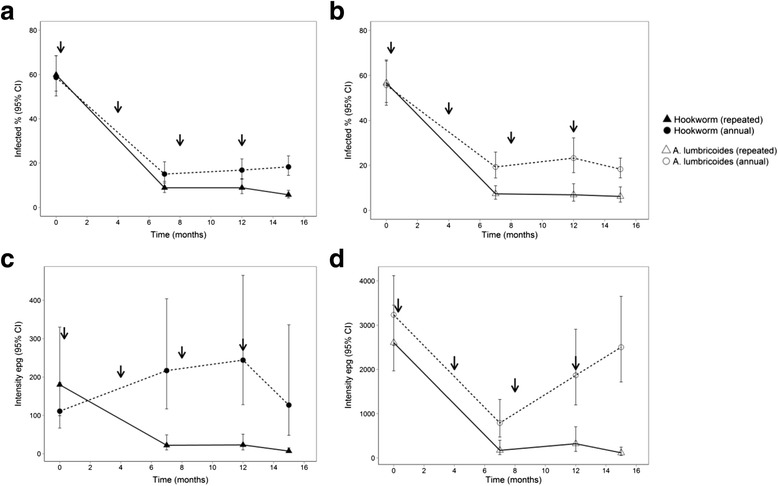
Proportion of infected children and mean intensity of hookworm (**a**, **c**) and *Ascaris lumbricoides* (**b**, **d** (adapted from Kepha et al. 2016) [15]. Treatment time points are indicated by arrows; the annual treatment group received only the first treatment dose

Baseline infection patterns, as well as children’s characteristics, were generally comparable between the two treatment groups (see Table 1). Fig. 2 shows the proportions of children infected with hookworm and *A. lumbricoides*, and the mean intensity of infections at baseline and at the 15-month follow-up point. *T. trichiura* infection was rare (1%) and therefore not included further in the analysis.

In the 4-monthly (repeated) treatment group, the proportion of children infected with hookworm decreased from 59.9 to 5.7%, while *A. lumbricoides* infections decreased from 55.7 to 6.2% (see Table 2). Interestingly, the last two anthelmintic treatments had only a slight effect on the prevalence and intensity of the two parasites (see Fig. 2). In the single treatment group, hookworm infections decreased from 58.7 to 18.3%, and *A. lumbricoides* infections from 67 to 23.3%. Residual hookworm and *A. lumbricoides* infections at the 15-month follow-up point were significantly higher in the single treatment group with an absolute difference of 12.6% (95% *CI*: 8.9–16.3%) for hookworm and 17.1% (95% *CI*: 13.1–21.1%) for *A. lumbricoides*. There were significant reductions in the mean intensity of hookworm and *A. lumbricoides* infections among children in the 4-monthly treatment group, but not in the single treatment group (see Table 2).

**Table 2:** Proportions of children infected with hookworm and *Ascaris lumbricoides,* and average intensity of infections at baseline and 15 months after treatment (*n* = 1141), by treatment group

	Proportion infected, % (95% *CI*)		Intensity of infection, epg (95% *CI*)	
Treatment group	Single	Four-monthly	Single	Four-monthly
Hookworm				
Baseline (Feb–Jun 2013)	58.7 (50.3–68.5)	59.9 (52.5–68.4)	111 (67–185)	180 (98–330)
15-month follow-up (Sept 2014)	18.3 (14.4–23.3)	5.7 (4.2–7.7)	126 (47–336)	7 (3–15)
Relative reduction (%)	68.8	90.5	Increase: 13.5	96.1
Odds ratio (95% *CI*)	0.09 (0.07–0.13)	0.02 (0.01–0.04)	0.59 (0.31–1.14)	0.03 (0.02–0.07)
*P* ^1^	<0.001	<0.001	0.116	<0.001
*A. lumbricoides*				
Baseline (Feb–Jun 2013)	56.7 (46.7–66.4)	55.7 (47.9–66.9)	3 234 (2 539–4 119)	2 606 (1 968–3 449)
15-month follow-up (Sept 2014)	23.3 (17.6–30.8)	6.2 (3.7–10.5)	2 504 (1 717–3 651)	117 (56–244)
Relative reduction (%)	58.9	88.7	22.6	95.5
Odds ratio (95% *CI*)	0.09 (0.06–0.13)	0.01 (0.01–0.03)	0.64 (0.57–1.10)	0.01 (0.01–0.04)
*P* ^1^	<0.001	<0.001	0.095	<0.001

^1^ *P*-values were obtained using mixed-effect logistic and negative binomial regression analyses with random intercepts for schools

### Predisposition to infections

The analysis demonstrated strong evidence for predisposition to hookworm and *A. lumbricoides* infections in all rounds of observations (all *P*-values < 0.001) (see Additional file 2: Table S2). Spearman’s rank correlation coefficients between all pairwise comparisons were 0.11–0.31 for hookworm and 0.24–0.57 for *A. lumbricoides*. Kendall’s Tau correlation coefficients were slightly lower.

### Factors associated with residual infection after four-monthly treatments

Results of the unadjusted analysis of factors associated with residual infections are shown in Tables 3 and 4. In the adjusted analysis, residual hookworm infection at the 15-month follow-up point was more common among boys and children who were underweight (see Table 3). The association with being underweight also remained when replacing mid-year age by the highest possible exact age, however, it was not observed when assuming the youngest possible exact age (see Table S3).

**Table 3:** Unadjusted and adjusted analyses for factors associated with hookworm infection at the 15-month follow-up point

		Proportion infected				Intensity of infection	
Variables	Categories	Odds ratio (95% *CI*)	*P*	Adjusted odds ratio^a^ (95% *CI*)	Adjusted *P*	Epg ratio (95% *CI*)	*P*
*Child characteristics*							
Sex	Male	1		1		1	
	Female	0.44 (0.20–0.97)	0.034	0.43 (0.20–0.97)	0.041	0.18 (0.03–1.00)	0.070
Age group	5–8	1		1		1	
	9–10	1.93 (0.64–5.82)		2.01 (0.66–6.07)		2.95 (0.26–32.67)	
	11–12	1.59 (0.50–5.01)	0.576	1.53 (0.48–4.83)	0.405	1.10 (0.10–12.36)	0.537
	13–15	2.02 (0.67–6.10)		1.88 (0.62–5.72)		4.19 (0.37–47.11)	
Anemia	Yes	0.56 (0.24–1.30)	0.162	0.50 (0.21–1.16)	0.095	1.83 (0.27–12.55)	0.628
Thin	Yes	1.81 (0.72–4.58)	0.235	1.66 (0.65–4.23)	0.318	5.21 (0.34–78.8)	0.152
Stunted	Yes	2.05 (1.00–4.20)	0.050	1.92 (0.93–3.96)	0.080	2.76 (0.20–350.5)	0.681
Underweight	Yes	8.46 (2.23–32.01)	0.005	5.50 (1.65–18.28)	0.006	2.87 (0.42–19.77)	0.258
Baseline infection							
Hookworm infection	Yes	2.02 (1.32–3.09)	0.001	1.63 (0.74–3.61)	0.225	0.79 (0.13–4.84)	0.804
Hookworm intensity	0	1		1		1	
	1–999	1.67 (0.75–3.72)	0.087	0.29 (0.08–1.14)	0.110	0.75 (0.13–4.48)	0.890
	> = 1 000	6.19 (1.48–28.88)		1		0.85 (0.00–217.17)	
*Household characteristics*							
Socioeconomic status	Poor	1		1		1	
	Less poor	0.97 (0.85–1.01)	0.608	0.97 (0.84–1.10)	0.593	1.08 (1.13–8.99)	0.734
Water source	Not covered	1		1		1	
	Covered	0.53 (0.12–2.40)	0.445	0.49 (0.11–2.26)	0.398	1.41 (0.10–160.42)	0.893
Floor	Mud	1		1		1	
	Cemented	0.97 (0.22–4.31)	0.977	0.97 (0.22–4.31)	0.970	0.34 (0.00–42.88)	0.700
Pit latrine	No	1		1		1	
	Yes	0.45 (0.19–1.08)	0.956	0.43 (0.18–1.05)	0.085	0.39 (0.03–5.73)	0.443
*School characteristics*							
Baseline school infection level							
Hookworm prevalence	<33	1		1		1	
	34–45	0.63 (0.23–1.72)	0.605	0.58 (0.21–1.61)	0.864	0.52 (0.06–4.94)	0.380
	>45%	0.96 (0.42–2.17)		0.91 (0.40–2.06)		1.94 (0.26–14.32)	
Hookworm epg	6–35	1		1		1	
	44–90	1.91 (0.81–4.48)	0.203	1.77 (0.75–4.18)	0.278	3.34 (0.43–26.04)	
	102–453	0.98 (0.37–2.62)		0.97 (0.36–2.59)		1.04 (0.12–2.62)	0.187
Latrine cleanliness	Clean	1		1		1	
	Dirty	0.40 (0.13–1.19)		1.77 (0.75–4.18)	0.127	0.74 (0.10–5.68)	
	Very dirty	1.27 (0.56–2.89)	0.110	0.97 (0.36–2.59)		4.32 (0.46–40.81)	0.341
Children per latrine	<50:1	1		1		1	
	>50:1	1.06 (0.51–2.18)	0.873	1.07 (0.52–2.22)	0.848	0.76 (0.32–1.80)	0.528
*Environmental characteristics*							
Elevation		1.00 (0.99–1.00)	0.300	1.00 (0.99–1.00)	0.344	1	
LST		0.73 (0.51–1.05)	0.101	1.09 (0.72–1.65)	0.670	0.75 (0.29–1.96)	0.564
NDVI	0.47–0.53	1		1		1	
	0.54–0.57	0.82 (0.33–2.03)	0.442	0.89 (0.36–2.21)	0.833	0.48 (0.06–4.16)	0.289
	0.58–0.67	1.20 (0.50–2.89)		1.15 (0.48–2.78)		1.78 (0.20–16.08)	
Distance from water bodies		1.08 (0.46–2.58)	0.854	1.10 (0.46–2.62)	0.831	0.60 (0.08–4.44)	0.615

*Abbreviations*: *NDVI* Normalized difference vegetation index, *LST* Land surface temperature

Elevation meters above sea level

^a^Adjusted for sex and hookworm baseline infection

**Table 4:** Unadjusted and adjusted analyses for factors associated with *A. lumbricoides* infection at the 15-month follow-up point

		Proportion infected				Intensity of infection	
Variable	Categories	Odds ratio (95% *CI*)	*P*	Adjusted^a^ odds ratio (95% *CI*)	Adjusted *P*	Epg ratio (95% *CI*)	*P*
*Child characteristics*							
Sex	Male	1		1		1	
	Female	0.79 (0.39–1.61)	0.510	0.79 (0.38–1.61)	0.510	0.65 (0.09–4.93)	0.683
Age group	5–8	1		1		1	
	9–10	1.06 (0.43–2.65)		1.07 (0.43–2.65)		0.53 (0.03–8.27)	
	11–12	0.66 (0.24–1.84)	0.781	0.66 (0.24–1.84)	0.752	0.18 (0.01–3.02)	0.650
	13–15	0.80 (0.29–2.23)		0.80 (0.29–2.24)		0.54 (0.03–9.30)	
Anaemia	Yes	1.23 (0.59–2.57)	0.572	1.42 (0.68–2.95)	0.354	2.85 (0.34–23.52)	0.328
Thin	Yes	1.53 (0.58–2.50)	0.703	1.48 (0.56–3.88)	0.441	1.33 (0.06–31.92)	0.859
Stunted	Yes	1.16 (0.54–1.51)	0.989	1.13 (0.52–2.42)	0.763	1.99 (0.21–18.49)	0.527
Underweight	Yes	0.74 (0.09–6.00)	0.766	0.62 (0.08–4.98)	0.628	7.51 (0.03–1923.72)	0.319
Baseline infection							
*A. lumbricoides* infection	Yes	4.37 (1.62–11.82)	0.001	3.66 (1.36–9.90)	0.010	2.36 (0.24–23.45)	0.467
*A. lumbricoides* intensity	0	1		1		1	
	1–4 999	3.77 (1.34–10.61)	0.003	0.62 (0.28–1.39)	0.259	2.88 (0.33–25.16)	0.617
	> = 5 000	6.02 (1.96–18.46)		1		2.78 (0.16–49.85)	
*Household characteristics*							
Socioeconomic status	Poor	1		1		1	
	Less poor	0.98 (0.86–1.11)	0.736	0.98 (0.86–1.11)	0.736	1.08 (0.13–8.99)	0.941
Water source	Not covered	1		1		1	
	Covered	0.61 (0.11–3.12)	0.570	0.61 (0.11–3.12)	0.570	1.64 (0.03–905.05)	0.883
Floor	Mud	1		1		1	
	Cemented	0.55 (0.07–4.39)	0.545	0.55 (0.07–4.39)	0.545	1.82 (0.03–130.16)	0.764
Pit latrine	No	1		1		1	
	Yes	1.75 (0.49–6.25)	0.359	1.75 (0.49–6.25)	0.359	2.20 (0.10–50.24)	0.656
*School characteristics*							
Baseline school infection level							
*A. lumbricoides* prevalence	<27%	1		1		1	
	28–30%	4.02 (1.47–14.55)		4.04 (1.08–15.16)		4.02 (0.34–47.00)	0.630
	>40%	0.60 (2.62–21.89)	0.019	0.60 (1.57–19.54)	0.011	5.61 (0.51–61.74)	
*A. lumbricoides* epg	366–913	1		1		1	
	914–2 497	2.00 (0.63–6.33)	0.095	2.00 (0.63–6.33)	0.095	1.26 (0.11–14.12)	0.878
	>2 497	3.48 (1.17–10.36)		3.38 (1.17–10.36)		2.27 (0.19–27.90)	
Latrine cleanliness	Clean	1		1		1	
	Dirty	0.53 (0.20–1.41)	0.049	0.48 (0.21–1.21)	0.030	0.17 (0.01–1.88)	0.446
	Very dirty	0.18 (0.04–0.86)		0.21 (0.05–0.92)		0.23 (0.02–3.09)	
Children per latrine	<50:1	1		1		1	
	>50:1	0.74 (0.27–2.04)	0.554	0.74 (0.27–2.04)	0.554	1.21 (0.16–9.11)	0.852
*Environmental characteristics*							
Elevation		1.04 (0.99–1.01)	0.402	1.01 (1.00–1.03)	0.040	1.00 (0.98–1.03)	0.991
LST (^o^C)	18.0–19.9	1		1		1	
	20.0–20.7	0.49 (0.21–1.12)	0.396	2.16 (0.62–7.55)	0.112	0.31 (0.03–3.63)	0.260
	20	0.21 (0.05–0.92)		3.08 (0.97–9.80)		1.23 (0.08–17.9)	
NDVI	0.47–0.53	1		1		0.02 (0.00–1.45)	0.761
	0.54–0.57	1.33 (0.34–5.21)	0.959	2.22 (0.71–6.92)	0.345		
	0.58–0.67	1.67 (0.43–6.51)		1.63 (0.63–4.23)			
Distance from water bodies		0.82 (0.40–1.70)	0.586	0.84 (0.24–2.99)	0.793	0.21 (0.00–22.94)	0.505

*Abbreviations*: *NDVI* Normalized difference vegetation index, *LST* Land surface temperature

Elevation meters above sea level

^a^Adjusted for sex and *A. lumbricoides* baseline infection, school-level *A. lumbricoides* baseline infection, and latrine cleanliness

Residual *A. lumbricoides* infection was positively associated with baseline individual infection status, baseline school-level infection prevalence, latrine cleanliness, and increasing elevation (see Table 4). No variables were found to be associated with residual infection intensity of any STH. A comparison of associations with residual infections among children in the single annual treatment group is provided in Additional file 2: Tables S4 and S5.

## Discussion

In line with the WHO strategy for controlling STH infections in endemic countries, the Kenya national school-based deworming program currently provides annual delivery of albendazole to school children [19]. Consistent with our understanding of the dynamics of transmission and control [14] and previous studies [20–22], we found that impacts on both prevalence and intensity of infection were significantly higher among children receiving 4-monthly treatments compared to those who received a single annual treatment. However, even after four repeated rounds of deworming in 15-month follow-up period a small proportion of children had residual hookworm and/or *A. lumbricoides* infection. This may reflect the maintenance of transmission among untreated population, especially adult populations or the failure to clear infections after treatment in a proportion of children [8, 23].

Our analysis also showed that children were highly predisposed to infections of both hookworm and *A. lumbricoides*, so the same children repeatedly acquire higher infection loads. Such correlations between pre- and post-treatment infection intensities are consistent with previous studies [24, 25], and may be a reflection of the increased exposure to infectious stages of a subgroup of children due to their behaviour or variations in environmental conditions (built and natural), as well as genetic or non-genetic physiological factors leading to higher susceptibility [24, 25]. Moreover, high baseline infection levels may also contribute to increased contamination of the children’s household and school environment [13, 26, 27]. Pre-treatment infection status was also the only variable associated with residual *A. lumbricoide*s infection after all four doses were administered. Therefore, the underlying mechanisms, which put children repeatedly at a higher risk of *A. lumbricoides* infections and hence reduce a successful treatment outcome, could not be clarified. For hookworm, however, associations with baseline infections were no longer significant after adjusting for sex.

The risk of hookworm infection at 15 months was higher among undernourished children assuming mid-year age or the highest possible exact age. A positive association between hookworm and undernourishment has also been reported by previous longitudinal studies [12, 26, 28]. This may suggest that wasted children either get reinfected more quickly or struggle to clear infection following treatment. Malnutrition and parasitic infections share a geographic distribution, mostly occurring among the poor, and it is therefore difficult to establish the causal pathway [11]. It is plausible that a helminth infection leads to malnutrition because physiological responses such as malabsorption or diarrhoea affect the ability of an individual to directly benefit from the nutrients ingested [29]. Undernutrition has also been demonstrated to impair immunity by depressing Th2 immune effectors and IgE, which may lead to increased risk of helminth infections [10]. An individual’s nutritional status may also directly influence treatment efficacy by, for example, altering drug absorption, metabolism, or uptake by the parasite. Reduced benzimidazole treatment efficacy has been previously described among malnourished individuals in animal models [10].

Previous studies demonstrate that children with adequate access to improved sanitation at the household and school levels were at a reduced risk of acquiring STH infections, and improved WASH conditions were positively associated with a higher impact of anthelmintic treatment [30–32]. Moreover, handwashing and access to soap was found to be associated with reduced odds for reinfection with STHs [31]. In this study, reinfection was not associated with any of the tested household WASH factors. This may be explained by the limited variability of access to improved WASH in the study area: overall, 95% of the households had access to an improved water source for drinking and 88% of households had access to a pit latrine, while no households had access to extremely improved latrines or flush toilets. As reported in previous studies, dirty pit latrines appeared to have a protective effect against hookworm infection. It has been suggested that school children should avoid dirty toilets, which may reduce their exposure to hookworm infections [18].

Our study had some limitations. As the children were originally selected for a clinical trial investigating the impact of deworming on the incidence of malaria, this secondary analysis was not taken into account during the study design and no margins of significant difference in STH infection were specified *a priori*. Presence of hookworm and *A. lumbricoides* was based on a single stool sample, and the technique used, Kato-Katz, has previously been demonstrated to have low diagnostic accuracy, especially when intensity of infection is low [33]. Therefore, it is possible that post-treatment results underrepresented the actual infection levels and low-intensity infections were missed. Additionally, we did not determine cure rates (clearance of infections after treatment), which makes it impossible to differentiate between reinfection or treatment failure. As we did not collect the exact birth dates of the children, baseline nutritional status was calculated based on age mid-point. Sensitivity analysis demonstrated that associations may change depending on the age assumption (in the extreme case, using the youngest possible exact age for each child). Therefore, although logistically difficult, our study highlights the importance of collecting exact birth dates. Collecting more detailed information at the household level may have been useful, such as the number of people living in a household and behaviours such shoe wearing and comprehensive household WASH. Not wearing shoes and overcrowding within households has been associated with helminth infection.

## Conclusions

Our results show that 4-monthly treatments were more effective than a single annual treatment. Repeated treatments dramatically reduced the intensity of infections, but failed to completely clear hookworm and *A. lumbricoides* infections among school children in Kenya. This suggests that increasing the treatment frequency among school children alone may not be sufficient to interrupt transmission in such a community. Such findings highlight the impact of periodic deworming for reducing the intensity of infections, but in order to reduce transmission in the community, the following should be emphasized: (i) the need for integrating deworming with interventions that help reduce exposure to infections such as access to improved WASH at both school and household levels, and (ii) the value of expanding treatment to the entire community.

## Additional files

Additional file 1: Multilingual abstracts in the five official working languages of the United Nations. (PDF 849 kb)
Additional file 2: (DOC 2410 kb)

## Abbreviations

BMIZ: Body mass index for age
*CI*: Confidence interval
epg: Eggs per gram
HAZ: Height for age
PCA: Principal component analysis
SD: Standard deviation
STH: Soil-transmitted helminth
WASH: Water, sanitation, and hygiene
WAZ: Weight for age
WHO: World Health Organization

## References

[1] Pullan RL, Smith JL, Jasrasaria R, Brooker SJ (2014). Global numbers of infection and disease burden of soil transmitted helminth infections in 2010. Parasit Vectors.

[2] Pullan RL, Brooker SJ (2012). The global limits and population at risk of soil-transmitted helminth infections in 2010. Parasit Vectors.

[3] Brooker S, Clements AC, Hotez PJ, Hay SI, Tatem AJ, Bundy DA (2006). The co-distribution of *Plasmodium falciparum* and hookworm among African schoolchildren. Malar J.

[4] Bundy DA (1986). Epidemiological aspects of Trichuris and trichuriasis in Caribbean communities. Trans R Soc Trop Med Hyg.

[5] Elkins DB, Haswell-Elkins M, Anderson RM (1986). The epidemiology and control of intestinal helminths in the Pulicat Lake region of Southern India. I. Study design and pre- and post-treatment observations on Ascaris lumbricoides infection. Trans R Soc Trop Med Hyg.

[6] Forrester JE, Scott ME, Bundy DA, Golden MH (1990). Predisposition of individuals and families in Mexico to heavy infection with Ascaris lumbricoides and Trichuris trichiura. Trans R Soc Trop Med Hyg.

[7] Upatham ES, Viyanant V, Brockelman WY, Kurathong S, Ardsungnoen P, Chindaphol U (1992). Predisposition to reinfection by intestinal helminths after chemotherapy in south Thailand. Int J Parasitol.

[8] Keiser J, Utzinger J (2008). Efficacy of current drugs against soil-transmitted helminth infections: systematic review and meta-analysis. JAMA.

[9] Anderson R, Truscott J, Hollingsworth TD (2014). The coverage and frequency of mass drug administration required to eliminate persistent transmission of soil-transmitted helminths. Philos Trans R Soc Lond B Biol Sci.

[10] Koski KG, Scott ME (2001). Gastrointestinal nematodes, nutrition and immunity: breaking the negative spiral. Annu Rev Nutr.

[11] Yap P, Utzinger J, Hattendorf J, Steinmann P (2014). Influence of nutrition on infection and re-infection with soil-transmitted helminths: a systematic review. Parasit Vectors.

[12] Halpenny CM, Paller C, Koski KG, Valdes VE, Scott ME (2013). Regional, household and individual factors that influence soil transmitted helminth reinfection dynamics in preschool children from rural indigenous Panama. PLoS Negl Trop Dis.

[13] Cundill B, Alexander N, Bethony JM, Diemert D, Pullan RL, Brooker S (2011). Rates and intensity of re-infection with human helminths after treatment and the influence of individual, household, and environmental factors in a Brazilian community. Parasitology.

[14] Anderson RM, Medley GF (1985). Community control of helminth infections of man by mass and selective chemotherapy. Parasitology.

[15] Kepha S, Nuwaha F, Nikolay B, Gichuki P, Mwandawiro CS, Mwinzi PN (2016). Effect of Repeated Anthelminthic Treatment on Malaria in School Children in Kenya: A Randomized, Open-Label. Equivalence Trial J Infect Dis.

[16] WHO (2007). Anthroplus: growth reference 5–19 years.

[17] Filmer D, Pritchett LH (2001). Estimating wealth effects without expenditure data-or tears: an application to educational enrollments in states of India. Demography.

[18] Freeman MC, Chard AN, Nikolay B, Garn JV, Okoyo C, Kihara J (2015). Associations between school- and household-level water, sanitation and hygiene conditions and soil-transmitted helminth infection among Kenyan school children. Parasit Vectors.

[19] Mwandawiro C, Nikolay B, Kihara JH, Ozier O, Mukoko DA, Mwanje MT (2013). Monitoring and evaluating the impact of national school-based deworming in Kenya: study design and baseline results. Parasit Vectors.

[20] Kirwan P, Asaolu SO, Molloy SF, Abiona TC, Jackson AL, Holland CV (2009). Patterns of soil-transmitted helminth infection and impact of four-monthly albendazole treatments in preschool children from semi-urban communities in Nigeria: a double-blind placebo-controlled randomised trial. BMC Infect Dis.

[21] Wiria AE, Hamid F, Wammes LJ, Kaisar MM, May L, Prasetyani MA (2013). The effect of three-monthly albendazole treatment on malarial parasitemia and allergy: a household-based cluster-randomized, double-blind, placebo-controlled trial. PLoS One.

[22] Kinungu’hi S, Magnussen P, Kishamawe C, Todd J, Vennervald BJ (2015). The impact of anthelmintic treatment intervention on malaria infection and anaemia in school and preschool children in Magu district, Tanzania: an open label randomised intervention trial. BMC Infect Dis.

[23] Anderson RM, Truscott JE, Pullan RL, Brooker SJ, Hollingsworth TD (2013). How effective is school-based deworming for the community-wide control of soil-transmitted helminths?. PLoS Negl Trop Dis.

[24] Anderson RM (1986). The population dynamics and epidemiology of intestinal nematode infections. Trans R Soc Trop Med Hyg.

[25] Schad GA, Anderson RM (1985). Predisposition to hookworm infection in humans. Science.

[26] Hesham Al-Mekhlafi M, Surin J, Atiya AS, Ariffin WA, Mohammed Mahdy AK, Che AH (2008). Pattern and predictors of soil-transmitted helminth reinfection among aboriginal schoolchildren in rural Peninsular Malaysia. Acta Trop.

[27] Brooker S, Clements AC, Bundy DA (2006). Global epidemiology, ecology and control of soil-transmitted helminth infections. Adv Parasitol.

[28] Papier K, Williams GM, Luceres-Catubig R, Ahmed F, Olveda RM, McManus DP (2014). Childhood malnutrition and parasitic helminth interactions. Clin Infect Dis.

[29] Hagel I, Lynch NR, Di Prisco MC, Perez M, Sanchez JE, Pereyra BN (1999). Helminthic infection and anthropometric indicators in children from a tropical slum: Ascaris reinfection after anthelmintic treatment. J Trop Pediatr.

[30] Ziegelbauer K, Speich B, Mausezahl D, Bos R, Keiser J, Utzinger J (2012). Effect of sanitation on soil-transmitted helminth infection: systematic review and meta-analysis. PLoS Med.

[31] Strunz EC, Addiss DG, Stocks ME, Ogden S, Utzinger J, Freeman MC (2014). Water, sanitation, hygiene, and soil-transmitted helminth infection: a systematic review and meta-analysis. PLoS Med.

[32] Jia TW, Melville S, Utzinger J, King CH, Zhou XN (2012). Soil-transmitted helminth reinfection after drug treatment: a systematic review and meta-analysis. PLoS Negl Trop Dis.

[33] Nikolay B, Brooker SJ, Pullan RL (2014). Sensitivity of diagnostic tests for human soil-transmitted helminth infections: a meta-analysis in the absence of a true gold standard. Int J Parasitol.

