# Supplementary material for: Impact of single annual treatment and four-monthly treatment for hookworm and Ascaris lumbricoides, and factors associated with residual infection among Kenyan school children
Source: Infect Dis Poverty. 2017 Feb 9;6:30. doi: 10.1186/s40249-017-0244-z (PMC5299645; doi:10.1186/s40249-017-0244-z)
Supplement: Additional file 1: — Multilingual abstracts in the five official working languages of the United Nations. (PDF 849 kb) [file 40249_2017_244_MOESM1_ESM.pdf]

Translation of the abstract into the five official working languages of the United Nations

أثر العلاج بالجرعة السنوية الوحيدة، والعلاج كل أربعة أشهر لمرض الدودة الخطافية، والاسكارس، والعوامل المرتبطة ببقايا المرض بين أطفال المدارس في كينيا

استيلا كيفا، وشارلس س. مواندوايرو، وروي م. أندرسون، وراشيل ل. بولان، وفريد نواها، وجور كانو، وسامي نجيني، وموريس ر. أوديري، واليزابث ألين، وسايمون ج. بروكر، وبيرقيت نيكولاي.

ملخّص:

معلومات أساسية: تُنفّذ حملات طرد الديدان بصورة واسعة في مختلف البلدان لتخفيف عبء الديدان الطفيلية المنقولة عن طريق التربة، بيد أن التواتر في تناول الأدوية يتفاوت من بيئة لأخرى. وفي هذه الدراسة عقدنا مقارنة بين أثر العلاج بالجرعة السنوية الوحيدة، والعلاج كل أربعة أشهر من خلال المتابعة بين أطفال المدارس الكينية، وتقصّى العوامل ذات الصلة ببقايا المرض.

الطُرُق: أجرينا تحليلاً ثانوياً من محاولة تقصّ عشوائية فيما إذا كان طرد الديدان الطفيلية المنقولة عن طريق التربة يقلل مخاطر الإصابة بالملاريا. وقد جرى تتبع الأطفال الذين تناولوا عقار البيندازول (عقار طارد للديدان) كل أربعة أشهر، لفترة طويلة استمرت من فبراير 2014 وحتى أكتوبر 2014. وجرى فحص الأثر النسبي للعلاج والعوامل المرتبطة ببقايا المرض باستخدام أساليب الانتكاسة متعددة الأثار. وقد جرى قياس قابلية الإصابة استناداً إلى معامل سبيرمان وكاندال لارتباط الرتب.

النتائج: في المجموعة التي تناولت العلاج كل أربعة أشهر، انخفضت نسبة الأطفال المصابين بالدودة الخطافية من 59,9% إلى 5,7%، بينما انخفض نسبة الإصابات بالاسكارس من 55,7% إلى 6,2%. أما في المجموعة التي تناولت العلاج بالجرعة السنوية الوحيدة في الفترة نفسها، فقد انخفضت الإصابات بالدودة الخطافية من 57,7% إلى 18,3% (أي بفرق مطلق للانخفاض بنسبة 12,6%، 95% CI:13,1-21,1%). وهناك قابلية قوية للإصابة بالنوعين من الديدان المعوية التي تعيش في التربة. وارتبطت الإصابة ببقايا مرض الدودة الخطافية بين الأطفال الذين تلقوا العلاج لأربعة أشهر بالأطفال الذكور، والوضع الغذائي الأساسي، في حين ارتبطت الإصابة بالاسكارس بالأفراد، والإصابة بالمستوى المدرسي الأساسي، وبنظافة المراحيض في المدارس.

الخلاصة: خلُصت الدراسة إلى أن تناول العلاج كل أربعة أشهر كان أكثر فاعلية من العلاج السنوي الوحيد. وقد أدى العلاج المتكرر إلى انخفاض ملحوظ في كثافة الديدان الطفيلية المنقولة عن طريق التربة، غير أنه لم يقض نهائياً على الإصابات في مدارس الأطفال في كينيا، وهو انعكاس افتراضي لإعادة الإصابة في البيئات التي يكون فيها انتشار مستمر للإصابة.

Translated from English version into Arabic by Hassan Adam, through

TRANSLATORS
WITHOUT BORDERS

每年一次和每四个月一次的钩虫、蛔虫的治疗对肯尼亚学校儿童的疗效以及残留感染的影响因素

Stella Kepha, Charles S. Mwandawiro, Roy M. Anderson, Rachel L. Pullan, Fred Nuwaha, Jorge Cano, Sammy M. Njenga, Maurice R. Odiere, Elizabeth Allen, Simon J. Brooker, Birgit Nikolay

摘要

引言:学校驱除寄生虫策略广泛应用于多个国家减少土源性寄生虫(STHs)的疾病负担。然而，对不同环境使用药物的评论也不同。本研究通过随访肯尼亚学校儿童比较每年一次和每四个月一次的治疗效果，并探究残余感染的相关影响因素。

方法:本研究对一个随机试验的数据进行了二次分析，并调查 STHs 的驱虫策能否改变患疟

疾的风险。2014 年 2 月至 10 月，对接受每年一次或每四个月一次阿苯哒唑治疗的儿童进行纵向随访。采用混合效应回归模型研究残存感染的影响因素与治疗相关的影响因素之间的关系。根据斯皮尔曼秩和检验和 Kendall’s Tau 相关系数评估易感性。
结果:在每四个月一次的治疗组，儿童钩虫感染率从 59.9%下降到 5.7%，而蛔虫感染从 55.7%下降到 6.2%。在每年一次的治疗组中，同一时期的钩虫感染从 58.7%减少到 18.3% (减少绝对差 12.6%, 95%*CI*: 8.9-16.3%)，而蛔虫的感染率从 56.7%减少到 23.3% (减少绝对差 17.1%, 95%*CI*:13.1-21.1%)。结果证明，所有的 STHs 均有显著的易感性。每四个月一次的治疗组中，儿童的钩虫残存感染与男性和营养基线水平有关，而蛔虫的感染与个人和学校感染基线水平、学校厕所清洁状况有关。
结论:本研究发现每四个月一次的治疗比每年一次的治疗更有效。重复治疗显著降低了 STHs 感染度，但肯尼亚学生儿童的感染的影响因素和流行区域的再感染的原因尚不明确。

Translated from English version into Chinese by Hong Tu, edited by Pin Yang, through

CHINESE CENTER FOR DISEASE CONTROL AND PREVENTION
NATIONAL INSTITUTE OF PARASITIC DISEASES

Impact du traitement annuel unique et du traitement quadrimestriel de l’ankylostome et des *Ascaris lumbricoides* et facteurs associés à l’infection résiduelle parmi des enfants kenyans en âge d’être scolarisés

Stella Kepha, Charles S. Mwandawiro, Roy M. Anderson, Rachel L. Pullan, Fred Nuwaha, Jorge Cano, Sammy M. Njenga, Maurice R. Odiere, Elizabeth Allen, Simon J. Brooker, Birgit Nikolay

Résumé

Contexte : la vermifugation à l’école est largement mise en œuvre dans de nombreux pays afin de réduire le fardeau des helminthes transmis par le sol (HTS), mais la fréquence d’administration du médicament varie néanmoins en fonction des circonstances. Dans le cadre de la présente étude, nous avons comparé l’impact d’un traitement annuel unique et d’un traitement quadrimestriel au cours d’une certaine durée de suivi parmi des enfants kenyans en âge d’être scolarisés et nous avons déterminé les facteurs associés à une infection résiduelle.
Méthodes : nous avons réalisé une analyse secondaire de données issues d’un essai randomisé afin de déterminer si l’administration d’un vermifuge contre les helminthes transmis par le sol (HTS) altérait le risque d’acquisition du paludisme. Les enfants ayant soit reçu un traitement unique ou un traitement quadrimestriel à base d’albendazole ont fait l’objet d’un suivi longitudinal de février 2014 à octobre 2014. L’impact relatif du traitement et les facteurs associés à des infections résiduelles ont fait l’objet d’un examen à l’aide de modèles de régression à effets mixtes. La prédisposition à l’infection a été évaluée sur la base du test de corrélation de Spearman et des coefficients de corrélation du tau de Kendall.
Résultats : au sein du groupe ayant bénéficié du traitement quadrimestriel, la proportion d’enfants infectés par l’ankylostome a diminué de 59,9 % à 5,7 %, tandis que les infections dues à l’*Ascaris lumbricoides* a chuté de 55,7 % à 6,2 %. Au sein du groupe ayant bénéficié d’un traitement unique, le taux d’infection due à l’ankylostome a diminué de 58,7 % à 18,3 % au cours de la même durée (12,6% de différence absolue en termes de réduction, *IC* à 95 % : 8,9–16,3 %), et celui de l’infection

due à l'*A. lumbricoides* a quant à lui chuté de 56,7 % à 23,3 % (17,1 % de différence absolue en termes de réduction, *IC* à 95 % : 13,1–21,1 %). Des preuves solides ont démontré l'existence d'une prédisposition aux deux types de HTS. L'ankylostomiase résiduelle parmi des enfants recevant un traitement quadrimestriel était associée au sexe masculin et à l'état nutritionnel de référence, tandis que l'infection due à l'*A. lumbricoides* était, elle, associée à l'infection individuelle et scolaire au départ ainsi qu'à la propreté des latrines dans les établissements scolaires.
Conclusions : cette étude a permis de déterminer qu'un traitement quadrimestriel s'avérait plus efficace qu'un traitement annuel unique. Des traitements répétés ont entraîné des réductions très importantes de l'intensité des infections par les HTS, mais n'ont néanmoins pas permis leur élimination complète parmi des enfants kenyans en âge d'être scolarisés, ce qui laisse supposer une réinfection dans un environnement impliquant une transmission continue.

Translated from English version into French by eric ragu, through

TRANSLATORS
WITHOUT BORDERS

Результаты лечения школьников Кении от анкилостом и *Ascaris lumbricoides* с периодичностью один раз в год и каждые четыре месяца и факторы, связанные с остаточной инфекцией

Стелла Кепха (Stella Kepha), Чарльз С. Мвандавиро (Charles S. Mwandawiro), Рой М. Андерсон (Roy M. Anderson), Рейчел Л. Пуллан (Rachel L. Pullan), Фред Нуваха (Fred Nuwaha), Хорхе Кано (Jorge Cano), Сэмми М. Ндженга (Sammy M. Njenga), Морис Р. Одиер (Maurice R. Odiere), Элизабет Аллен (Elizabeth Allen), Саймон Дж. Брукер (Simon J. Brooker), Биргит Николаи (Birgit Nikolay)

Аннотация

Краткое описание. Проведение дегельминтизации в школах для уменьшения заболеваемости геогельминтами широко распространено во многих странах, однако частота приёма лекарств отличается в разных местах. В данном исследовании мы сравнили результаты лечения кенийских школьников один раз в год и каждые четыре месяца с последующим наблюдением и изучили факторы, связанные с остаточной инфекцией.
Методы. Мы провели вторичный анализ данных испытания методом случайной выборки для изучения влияния дегельминтизации против геогельминтов на риск заболевания малярией. За детьми, получившими одноразовое лечение альбендазолом или лечение каждые четыре месяца, наблюдали по долготе с февраля по октябрь 2014 года. Относительное воздействие лечения и факторы, связанные с остаточной инфекцией, были изучены с использованием регрессионных моделей со смешанными эффектами. Предрасположенность к инфицированию оценивалась на основе коэффициентов корреляции Спирмена и тау Кендалла.

Результаты. В группе, получившей лечение каждые четыре месяца, процент детей, заражённых анкилостомами, снизился с 59,9% до 5,7%; при этом заболеваемость *Ascaris lumbricoides* упала с 55,7% до 6,2%. В группе, получившей одноразовое лечение, заражение анкилостомами за тот же период времени снизилось с 58,7% до 18,3% (абсолютная разница в снижении 12,6%, 95%*CI*: 8,9–16,3%) и заражение *A. lumbricoides* с 56,7% до 23,3% (абсолютная разница в снижении 17,1%, 95%*CI*: 13,1–21,1%). Убедительные доказательства продемонстрировали предрасположенность к обоим типам геогельминтов. Остаточное инфицирование анкилостомами среди детей, получивших лечение каждые четыре месяца, было связано с мужским полом и базовым состоянием питания, в то время как инфицирование *A. lumbricoides* было связано с инфекцией на индивидуальном и общешкольном уровнях в начале исследования, а также чистотой туалетов в школах.
Заключение. Данное исследование показало, что лечение каждые четыре месяца было более эффективным, чем одно лечение в год. Повторные применения препарата привели к резкому снижению интенсивности заражения геогельминтами, однако полностью не устранили заболеваемость среди школьников в Кении, что возможно является следствием повторного заражения в среде, где происходит постоянная передача инфекции.

Translated from English version into Russian by Natalia Potashnik, through

TRANSLATORS WITHOUT BORDERS

Impacto del tratamiento anual único y del tratamiento cuatrimestral para la anquilostomiasis y *Ascaris lumbricoides* y factores asociados con la infección residual entre escolares kenianos

Stella Kepha, Charles S. Mwandawiro, Roy M. Anderson, Rachel L. Pullan, Fred Nuwaha, Jorge Cano, Sammy M. Njenga, Maurice R. Odiere, Elizabeth Allen, Simon J. Brooker, Birgit Nikolay

Resumen
Antecedentes: La desparasitación en la escuela se implanta ampliamente en varios países para reducir la carga de helmintos transmitidos por el suelo (STH). Sin embargo, la frecuencia de administración de fármacos varía en diferentes entornos. En este estudio, comparamos el impacto de un solo tratamiento anual y un tratamiento cuatrimestral en un seguimiento entre escolares kenianos, e investigamos los factores asociados con la infección residual.
Métodos: Se realizó un análisis secundario de los datos de un ensayo aleatorio que investigaba si la desparasitación de STH altera el riesgo de adquirir la malaria. Los niños que recibieron un solo tratamiento o tratamientos cuatrimestrales de albendazol fueron seguidos longitudinalmente desde febrero de 2014 hasta octubre de 2014. El impacto relativo del tratamiento y los factores asociados con infecciones residuales fueron investigados usando modelos de regresión de efectos mixtos. La predisposición a la infección se evaluó sobre la base del rango de Spearman y los coeficientes de correlación de Kendall Tau.

Resultados: En el grupo de tratamiento cuatrimestral, la proporción de niños infectados por anquilostomas disminuyó del 59,9% al 5,7%, mientras que las infecciones por *Ascaris lumbricoides* cayeron de 55,7% a 6,2%. En el grupo de tratamiento único, las infecciones por anquilostomas disminuyeron durante el mismo período de tiempo de 58,7% a 18,3% (12,6% de diferencia absoluta en la reducción, IC del 95%: 8,9-16,3%) y por *A. lumbricoides* del 56,7% al 23,3% (17,1 % de diferencia absoluta en la reducción, IC del 95%: 13,1-21,1%). Hubo indicios sólidos de predisposición a ambos tipos de STH. La infección por anquilostoma residual entre los niños en tratamiento cuatrimestral se asoció con el sexo masculino y un estado nutricional base, mientras que la infección por *A. lumbricoides* se asoció con la infección individual y escolar base, la limpieza de las letrinas en las escuelas.

Conclusiones: Este estudio concluyó que el tratamiento cuatrimestral era más eficaz que el tratamiento único anual. Los tratamientos repetidos llevaron a drásticas reducciones en las intensidades de STH, pero no erradicaron completamente las infecciones entre los escolares en Kenia, reflejo de reinfección en un entorno donde hay transmisión en curso.

Translated from English version into Spanish by Amparo Muñoz, through

TRANSLATORS
WITHOUT BORDERS
