# Supplementary material for: Impact of single annual treatment and four-monthly treatment for hookworm and Ascaris lumbricoides, and factors associated with residual infection among Kenyan school children
Source: Infect Dis Poverty. 2017 Feb 9;6:30. doi: 10.1186/s40249-017-0244-z (PMC5299645; doi:10.1186/s40249-017-0244-z)
Supplement: Additional file 2: — (DOC 2410 kb) [file 40249_2017_244_MOESM2_ESM.doc]

**Additional file 2**

**Index**

1. Generation of socioeconomic and toilet cleanliness indices 1

2. Sources and processing of environmental data 2

3. Predisposition to infection- pairwise correlations 5

4. Comparison of factors associated with residual infection between treatment groups 5

5. References 10

1. **Generation of socioeconomic and toilet cleanliness indices**

At enrollment, a household questionnaire was administered to parents/guardians to collect information on wall, floor, and roof construction materials, source of fuel, mobile phone ownership, and level of education of the household head. These factors were used to generate a wealth index based on principal component analysis (PCA) as previously described [1]. PCA is used to reduce a correlated set of variables to uncorrelated components, where each component is estimated through linear weighted combination of the initial variables [2].PCA scores were predicted based on the first component, which explained 53% of the variance. Subsequently, the score was divided into two groups (poor and less poor) based on the median (Table S1). The loadings of the first PCA component are summarised in Table S1.

School-level data on water, sanitation and hygiene (WASH) were collected by interviewing the head teacher or deputy head teacher and by visual inspection, using questionnaires and checklists previously used in Kenya. Conditions of school sanitation facilities were assessed based on observed ‘cleanliness’ status of the latrine, presence of visible faeces, excessive smell, and excessive flies combined by PCA (Table S1) and scores were predicted based on the first component which explained 60% of the variance. The loadings of the first PCA component are summarised in Table S1. Predicted scores were used to generate two categories (clean and not clean) based on the median.

| **Table S1** PCA loadings (first component) and weights for household socio economic index and toilet cleanliness indices**.** | | |
| --- | --- | --- |
| **Characteristic** | **PCA loading** | **Weight** |
| **Household socio economic index** |  |  |
| Material of wall construction | -0.099 | 0.01 |
| Material of roof construction | 0.662 | 0.44 |
| Type of fuel used for cooking | -0.676 | 0.46 |
| Net use | 0.169 | 0.03 |
| Level of education of household head | 0.259 | 0.07 |
| **Toilet cleanliness index** |  |  |
| Presence of smell | 0.499 | 0.25 |
| Pit full | 0.043 | 0.00 |
| Visible feces | 0.613 | 0.38 |
| Flies Present | 0.612 | 0.37 |

1. **Sources and processing of environmental data**

Elevation data at 250m resolution were derived from gridded digital elevation models (DEM) produced by the Shuttle Radar Topography Mission (SRTM). A gridded map of the aridity index, which is a generalized function of precipitation, temperature, and/or potential evapotranspiration, was obtained at 1 km^2^ resolution from CGIAR-CSI [3]. Averaged long-term enhanced vegetation index (EVI) for the period 2000 to 2013 was obtained from the Africa Soil Information Service (AfSIS) [4]. This vegetation index is collected at a 16-day basis by the *Moderate Resolution Imaging Spectroradiometer* (MODIS) sensor and delivered in monthly average raster datasets at 250m resolution by the Columbia University International Research Institute for Climate and Society (IRI).

Land surface temperature (LST) and normalized difference vegetation index (NDVI) were estimated from a satellite image of the study area obtained through the Landsat-8 Operational Land Imager (OLI) sensor at September, 2014. A level-1 product was downloaded from the U.S. Geological Survey (USGS) website [5] (Figure S1). In order to increase the spatial resolution of multispectral bands (provided at 30m resolution), a pan-sharpening process was applied, except for thermal infrared bands, using the 15-m panchromatic band. Atmospheric correction was then conducted for the bands 1 to 8 using the DOS model-based algorithm and assuming 1% minimum reflectance [6]. Radiance and ground reflectance were subsequently obtained for each band [7]. The thermal infrared band (TIR) 10 (10.60 - 11.19 µm) was used to estimate the effective at-sensor brightness temperature (
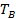
) using the Plank’s inverse functions as detailed elsewhere [8]. The final LST was estimated by the following equation:


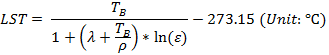


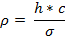


where,
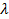
 is the wavelength of the emitted radiance which is equal to 10.8 µm;
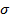
 is the Stefan Boltzmann’s constant which is equal to 5.67 x 10^-8^ Wm^-2^ K^-4^;
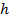
 is the Plank’s constant (6.626x10^-34^ JSec);
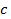
is the velocity of light (2.998x10^8^ m/sec) and
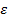
 is the spectral emissivity which was estimated as follows:


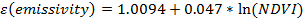


The spectral bands 4 (red: 0.64-0.67 µm) and 5 (NIR: 0.85-0.88) were used to generate NDVI of the study area (Figure S1).

| **Figure S1**. Land surface temperature and normalized difference vegetation index satellite image of the study area obtained through the Landsat-8 Operational Land Imager (OLI) sensor at September, 2014 |
| --- |
| **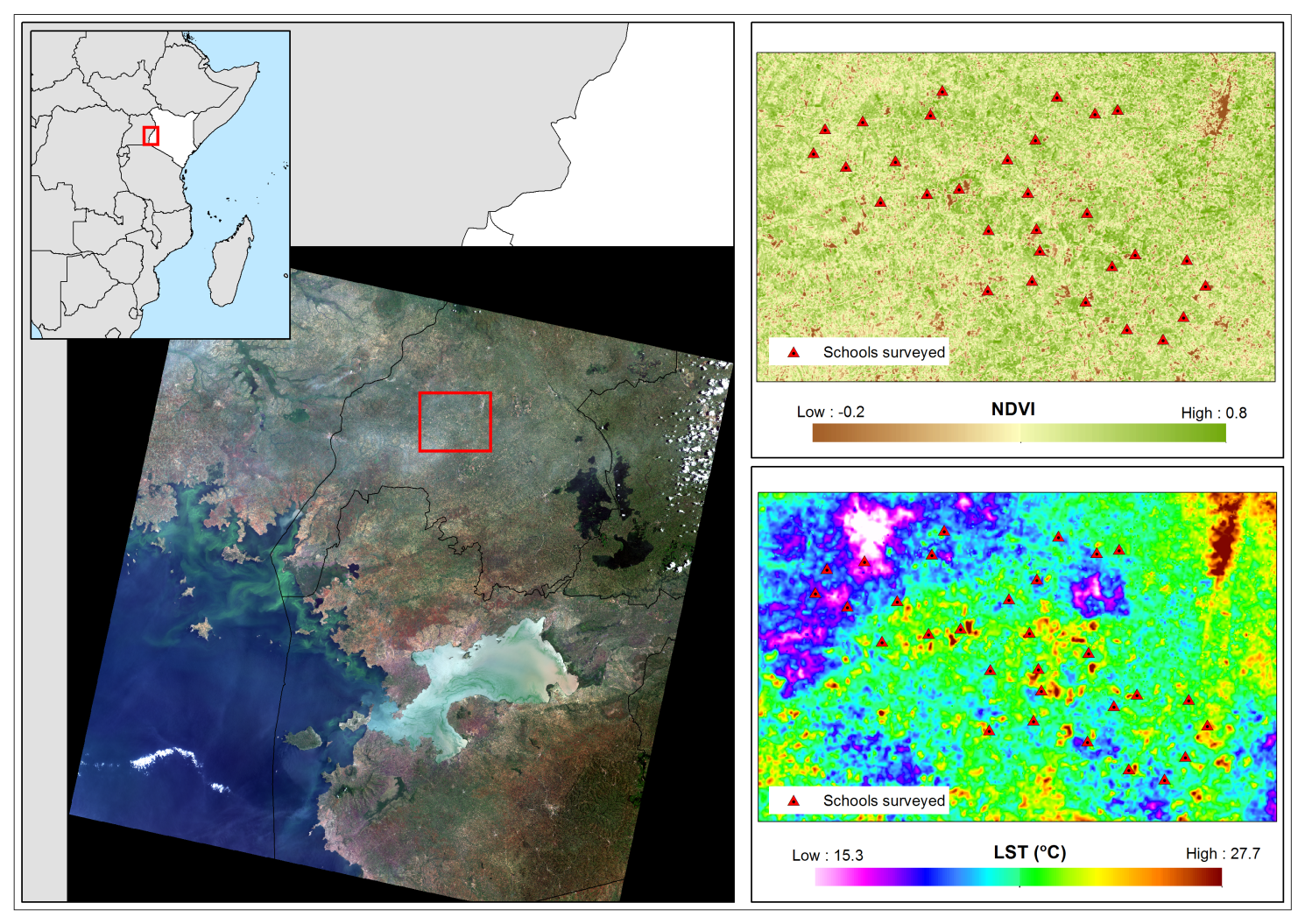** |

1. **Predisposition to infection- pairwise correlations**

| **Table S2.** Correlation coefficients in pairwise comparisons of infection intensities at baseline (Bl), 7 months (7m), 12 months (12m), and 15 months (15m) surveys. All p-values of Spearman’s and Kendall’s tau test were <0.001 strongly rejecting the null hypothesis of no predisposition. | | | | | | | | | | | | |  | |
| --- | --- | --- | --- | --- | --- | --- | --- | --- | --- | --- | --- | --- | --- | --- |
| **Comparison** | | **Bl-7m** | **Bl-12m** | | **Bl-15m** | | **7m-12m** | | **7m-15m** | | **12m-15m** | |  | |
| **Hookworm** | | | | | | | | | | | | | |  |
| **Spearman’s**  **(ρ)** | | 0.11 | 0.17 | | 0.15 | | 0.23 | | 0.14 | | 0.31 | |  | |
| **Kendall’s tau**  **(τ)** | | 0.10 | 0.15 | | 0.13 | | 0.22 | | 0.13 | | 0.29 | |  | |
| **A. lumbricoides** | | | | | | | | | | | | | |  |
| **Spearman’s**  **(ρ)** | | 0.29 | 0.30 | | 0.24 | | 0.46 | | 0.42 | | 0.57 | |  | |
| **Kendall’s tau**  **(τ)** | | 0.25 | 0.25 | | 0.20 | | 0.43 | | 0.40 | | 0.53 | |  | |
|  |  | | |  | |  | |  | |  | |  | | |
|  |  | | |  | |  | |  | |  | |  | | |
|  |  | | |  | |  | |  | |  | |  | | |

1. **Comparison of factors associated with residual infection between treatment groups**

To investigate differences in risk factors for residual infections according to administered treatment pressure or the allowed time for reinfection, we further quantified associations with residual infections by treatment group. The outcomes of this analysis were the proportion infected with hookworm and *A. lumbricoides* and the intensities of infection at the last follow-up survey (15 months after baseline assessment) including children from both treatment groups. Variables associated with residual infections in both treatment groups were first investigated using mixed effects regression models adjusting for treatment group and baseline individual infection (infection status for logistic regression and intensity of infection for negative binomial regression) and using a model selection procedure as described in the main methods section. Subsequently, we assessed differences in associations by treatment group by including interaction terms between the treatment group and each variable that remained significant in adjusted models. Interaction terms were subsequently removed in a backwards approach starting with the interaction terms with highest P-values and interaction terms were retained in the final model if the P-value of interaction was <0.25. Treatment group-specific odds ratios (OR) were estimated directly from the mixed effects regression models.

In the adjusted analysis of both treatment groups combined, residual hookworm infection at 15 months was more common among children who were underweight and infected with hookworm at baseline. Residual hookworm intensity was higher among boys, among thin children and those living at lower elevation (Table S3). Residual *A. lumbricoides* infection was overall positively associated with baseline infection status and there was an evident dose response of school baseline prevalence.

Some of the observed associations varied according to treatment group (Table S4). The odds ratio (OR) comparing underweight and normal weight children was more than twice as high among those who received repeated treatment, even though the test for effect modification was not statistically significant (interaction P=0.220). Moreover, the negative association of residual hookworm infection with elevation was stronger among children who received a single annual treatment (interaction P=0.100). For *A. lumbricoides* infection, the effect of school baseline infection prevalence on follow-up prevalence was more markedly among children who received repeated treatment (again interaction was not statistically significant with interaction P=0.194), while baseline infection status was positively associated with *A. lumbricoides* irrespective of the treatment group (interaction P=0.755).

**Table S3. Comparison of hookworm infection and baseline nutritional status by youngest possible exact age, mid- year age and oldest possible**

| **Characteristic** | **Youngest possible age** |  | **Mid-year age** |  | **Oldest possible age** |  |
| --- | --- | --- | --- | --- | --- | --- |
| Thin | 22 (16.2) |  |  |  | 24 (17.7) | 0.161 |
| Not thin | 114 (83.8) |  |  |  | 112 (86.3) |  |
| Stunted | 27 (19.9) | 0.185 | 98 (72.1) | 0.849 | 82 (60.3) | 0.402 |
| Not stunted | 109 (80.1) |  | 38 (27.9) |  | 54 (39.7) |  |
| Underweight | 8 (12.9) | 0.127 | 9 (14.5) | 0.004 | 44 (71.0) | <0.001 |
| Not under-weight | 54 (87.1) |  | 53 (85.5) |  | 18 (29.0) |  |
|  |  |  |  |  |  |  |
|  |  |  |  |  |  |  |

| **Table S4. Adjusted analysis for factors associated with hookworm and *A. lumbricoides* infection at 15 months in both treatment groups.** | | | | | | | | |
| --- | --- | --- | --- | --- | --- | --- | --- | --- |
|  |  | **Hookworm** | | | |  | ***Ascaris lumbricoides*** | |
|  |  | **Proportion infected^1^** | | **Intensity^2^** | |  | **Proportion infected^3^** | |
| Variable | Categories | Adjusted Odds ratio (95%CI) | Adjusted P-value | Adjusted epg ratio (95%CI) | Adjusted P-value |  | Adjusted Odds ratio 95%CI) | Adjusted P-value |
| ***Child characteristics*** |  |  |  |  |  |  |  |  |
| Sex | Boys | 1 |  | 1 |  |  | 1 |  |
|  | Girls | 0.91 (0.62-1.32) | 0.608 | 0.11 (0.07-0.40) | 0.010 |  | 1.04 (0.72-1.49) | 0.837 |
| Age group | 5-8 | 1 |  | 1 |  |  | 1 |  |
|  | 9-10 | 1.01 (0.58-1.75) |  | 2.24 (0.68-7.35) |  |  | 0.65 (0.40-1.06) |  |
|  | 11-12 | 1.37 (0.85-2.53) | 0.357 | 1.11 (0.32-3.93) | 0.462 |  | 0.71 (0.43-1.18) | 0.336 |
|  | 13-15 | 1.48 (0.80-2.36) |  | 2.05 (0.60-7.00) |  |  | 0.75 (0.45-1.24) |  |
| Anemia | Yes | 1.02 (0.68-1.52) | 0.931 | 1.23 (0.44-3.42) | 0.685 |  | 1.15 (0.79-1.68) | 0.471 |
| Thin | Yes | 1.39 (0.83-2.35) | 0.215 | 5.89 (1.58-21.9) | 0.008 |  | 1.35 (0.79-2.30) | 0.286 |
| Stunted | Yes | 0.86 (0.56-1.33) | 0.496 | 1.67 (0.60-4.63) | 0.319 |  | 1.00 (0.67-1.51) | 0.989 |
| Underweight | Yes | 2.78 (1.21-6.34) | 0.015 | 2.76 (0.26-28.89) | 0.320 |  | 0.58 (0.19-1.76) | 0.307 |
| Baseline infection |  |  |  |  |  |  |  |  |
| Hookworm infection | Yes | 2.02 (1.32-3.09) | 0.001 | 0.64 (0.26-1.61) | 0.338 |  | N/A |  |
| Hookworm intensity | 0 |  |  |  |  |  |  |  |
|  | 1-999 |  |  |  |  |  |  |  |
|  | >=1000 |  |  |  |  |  |  |  |
| *A. lumbricoides* infection | Yes | N/A |  | N/A |  |  | 3.55 (2.52-4.99) | **<**0.001 |
| *A. lumbricoides* intensity | 0 |  |  |  |  |  |  |  |
|  | 1-4999 |  |  |  |  |  |  |  |
|  | >=5000 |  |  |  |  |  |  |  |
| Study arm | Annual | 1 |  | 1 |  |  | 1 |  |
|  | Repeated | 0.25 (0.17-0.39) | <0.001 | 0.08 (0.03-0.23) | **<**0.001 |  | 0.19 (0.13-0.29) | <0.001 |
| ***Household characteristics*** | | | | | | | | |
| Socioeconomic status | Poor | 1 |  | 1 |  |  | 1 |  |
|  | Less poor | 0.94 (0.88-1.01) | 0.09 | 1.04 (1.44-20.76) | 0.610 |  | 0.71 (0.48-1.06) | 0.087 |
| Water source | Non improved  Source | 1 |  | 1 |  |  | 1 |  |
|  | Improved source | 0.72 (0.33-1.56) | 0.853 | 0.69 (0.10-4.89) |  |  | 0.89 (0.42-1.90) | 0.765 |
| Floor | Mud | 1 |  | 1 |  |  | 1 |  |
|  | Cemented | 1.08 (0.47-2.50) | 0.853 | 0.40 (0.05-3.76) | 0.464 |  | 0.54 (0.20-1.44) | 0.188 |
| Pit latrine | Absent | 1 |  | 1 |  |  | 1 |  |
|  | Present | 0.78 (0.45-1.35) | 0.382 | 0.41 (0.08-1.99) | 0.695 |  | 0.84 (0.48-1.47) | 0.554 |
| ***School characteristics*** | | | | | | | | |
| Baseline school infection level | |  |  |  |  |  |  |  |
| Hookworm prevalence | <33 | 1 |  | 1 |  |  |  |  |
|  | 34-45 | 0.71 (0.42-1.21) | 1.00 | 1.32 (0.42-4.13) | 0.842 |  | N/A |  |
|  | >45% | 0.53 (0.26-1.07) |  | 1.00 (0.34-2.95) |  |  |  |  |
| *A. lumbricoides* prevalence | <27% |  |  |  |  |  | 1 |  |
|  | 28-30% | N/A |  | N/A |  |  | 4.62 (1.47-14.55) |  |
|  | >40% |  |  |  |  |  | 7.58 (2.62-21.89) | 0.0007 |
| Hookworm epg | 6-35 | 1 |  | 1 |  |  |  |  |
|  | 44-90 | 0.99 (0.55-1.80) |  | 1.22 (0.39-3.77) |  |  |  |  |
|  | 102-453 | 0.97 (0.55-1.71) | 1.00 | 0.83 (0.27-2.62) | 0.781 |  |  |  |
| *A. lumbricoides* epg | 366-913 |  |  |  |  |  | 1 |  |
|  | 914-2497 | NA |  | N/A |  |  | 1.01 (0.53-1.95) | 0.932 |
|  | >2497 |  |  |  |  |  | 0.88 (0.35-2.22) |  |
| Latrine cleanliness |  |  |  |  |  |  | 1 |  |
|  | Clean | 1 |  | 1 |  |  | 0.83 (0.49-1.41) | 0.795 |
|  | Dirty | 0.91 (0.50-1.65) |  | 0.78 (0.28-2.19) |  |  | 0.90 (0.52-1.54) |  |
|  | Very dirty | 0.71 (0.41-1.23) | 0.453 | 2.11 (0.71-6.28) | 0.240 |  | 1 |  |
| Children per latrine | <50:1 | 1 |  | 1 |  |  | 0.93 (0.59-1.48) | 0.772 |
|  | >50:1 | 0.94 (0.63-1.41) | 0.766 | 0.76 (0.32-1.80) | 0.537 |  |  |  |
| ***Environmental characteristics*** | | | | | | | | |
| Elevation | 1^st^ tertile | 1 |  | 1 |  |  | 1 |  |
|  | 2^nd^ tertile | 0.57 (0.37-0.90) | 0.021 | 1.79 (0.65-4.89) | 0.105 |  | 1.51 (0.62-2.14) | 0.899 |
|  | 3^rd^ tertile | 0.53 (0.33-0.85) |  | 0.52 (0.18-1.51) |  |  | 1.08 (0.58-1.99) |  |
| LST |  | 1.28 (0.87-1.89) | 0.206 | 1.02 (0.58-1.80) | 0.935 |  | 1.14 (0.81-1.60) | 0.444 |
| NDVI |  | 0.50 (0.0-51.12) | 0.772 | 0.90 (0-22-2.73) | 0.983 |  | 0.12 (0.0-50.5) | 0.490 |
| Distance from water bodies |  | 0.97 (0.60-1.56) | 0.908 | 0.95 (0.24-3.70) | 0.945 |  | 0.82 (0.40-1.70) | 0.586 |
| Abbreviations: NDVI Normalized difference vegetation index; LST Land surface temperature; Elevation meters above sea level SD, standard deviation; WAZ, weight-for-age z-score; HAZ, height-for-age z-score; BMIZ, body mass index z-score; STH, soil-transmitted helminth; CI, confidence interval  ^1^ adjusted for underweight, hookworm infection and elevation  ^2^ adjusted for sex and being thin  ^3^ adjusted for school level baseline infection and baseline infection | | | | | | | | |

| **Table S5. Effect modification of treatment group on factors associated with hookworm and *A. lumbricoides* infection at 15 months** | | | | | | |
| --- | --- | --- | --- | --- | --- | --- |
| **Variable** | | **Annual treatment** | | **Repeated treatment** | | Effect modification  P value |
|  |  | Adjusted Odds ratio / epg ratio (95% CI) | Adjusted P value | Adjusted Odds ratio/ epg ratio (95% CI) | Adjusted P value |  |
| **Hookworm infection^1^** |  |  |  |  |  |  |
| Under weight | Yes | 1.82 (0.61-5.34) | 0.280 | 5.50 (1.56-17.25) | 0.007 | 0.220 |
| Hookworm infection | Yes | 2.04 (1.33-3.12) | 0.001 | 1.91 (0.85-4.29) | 0.120 | 0.901 |
| Elevation | 1244-1299 m | 1 |  | 1 |  |  |
|  | 1300-1350 m | 0.58 (0.37-0.90) | 0.001 | 1.14 (0.52-2.49) | 0.328 | 0.100 |
|  | 1351- 1392 m | 0.53 (0.33-0.86) |  | 0.52 (0.18-1.49) |  |  |
| **Hookworm infection intensity** | | | | | | |
| **Sex** | Female | 0.13 (0.04-0.40) | 0.004 | 0.19 (0.06-0.61) | 0.005 | 0.821 |
| Thin | Yes | 6.46 (0.99-42.02) | 0.051 | 5.07 (0.79-32.5) | 0.086 | 0.858 |
| ***A. lumbricoides infection*^2^** |  |  |  |  |  |  |
| *A. lumbricoides infection* | Yes | 2.71 (1.67-4.39) | <0.001 | 3.22 (1.19-8.68) | 0.021 | 0.755 |
| School level baseline *A. lumbricoides* infection | <27% | 1 |  | 1 |  |  |
|  | 28-30% | 1.12 (0.58-2.21) |  | 4.02 (1.04-12.5) | 0.043 | 0.94 |
|  | >40% | 2.67 (1.45-4.92) |  | 6.40 (1.75-23.32) |  |  |
| ^1^The final model was fitted with an interaction term between treatment group and underweight and elevation and treatment group  ^2^The final model was fitted with an interaction term between treatment group and baseline school level infection.  Abbreviations: CI, confidence interval | | | | | | |

1. **References**
